# Predicting miRNA-disease associations based on graph attention network with multi-source information

**DOI:** 10.1186/s12859-022-04796-7

**Published:** 2022-06-21

**Authors:** Guanghui Li, Tao Fang, Yuejin Zhang, Cheng Liang, Qiu Xiao, Jiawei Luo

**Affiliations:** 1 School of Information Engineering, East China Jiaotong University, Nanchang, China; 2 School of Information Science and Engineering, Shandong Normal University, Jinan, China; 3 College of Information Science and Engineering, Hunan Normal University, Changsha, China; 4 College of Computer Science and Electronic Engineering, Hunan University, Changsha, China

**Keywords:** miRNA-disease associations, Graph attention network, Feature fusion, Random forest

## Abstract

**Background:**

There is a growing body of evidence from biological experiments suggesting that microRNAs (miRNAs) play a significant regulatory role in both diverse cellular activities and pathological processes. Exploring miRNA-disease associations not only can decipher pathogenic mechanisms but also provide treatment solutions for diseases. As it is inefficient to identify undiscovered relationships between diseases and miRNAs using biotechnology, an explosion of computational methods have been advanced. However, the prediction accuracy of existing models is hampered by the sparsity of known association network and single-category feature, which is hard to model the complicated relationships between diseases and miRNAs.

**Results:**

In this study, we advance a new computational framework (GATMDA) to discover unknown miRNA-disease associations based on graph attention network with multi-source information, which effectively fuses linear and non-linear features. In our method, the linear features of diseases and miRNAs are constructed by disease-lncRNA correlation profiles and miRNA-lncRNA correlation profiles, respectively. Then, the graph attention network is employed to extract the non-linear features of diseases and miRNAs by aggregating information of each neighbor with different weights. Finally, the random forest algorithm is applied to infer the disease-miRNA correlation pairs through fusing linear and non-linear features of diseases and miRNAs. As a result, GATMDA achieves impressive performance: an average AUC of 0.9566 with five-fold cross validation, which is superior to other previous models. In addition, case studies conducted on breast cancer, colon cancer and lymphoma indicate that 50, 50 and 48 out of the top fifty prioritized candidates are verified by biological experiments.

**Conclusions:**

The extensive experimental results justify the accuracy and utility of GATMDA and we could anticipate that it may regard as a utility tool for identifying unobserved disease-miRNA relationships.

## Background

MicroRNAs (miRNAs) are short non-coding RNA molecules regulating the expression of other genes in biological processes and forming eukaryotic cell-dependent gene expression programs [1]. In 1993, Lee et al. [2] discovered the first miRNA in elegans. Subsequently, many researchers turned their focused on the role of miRNAs. Recently, several investigations reveal that differential expression of miRNAs is associated to the occurrence and progression of human diseases [3–5]. For instance, the first study for discovering the association between miRNA and cancer was published by Calin et al. [6], and the study showed that miR-15 has a significant inhibitory level relationship with chronic lymphocytic leukemia. In addition, biological experiments showed that miR-15 serves as an oncogene in lymphoma [7]. A further example of disease-miRNA relationships is miR-27b and miR-23b in breast cancer [8]. The impact of miR-27b and miR-23b in breast cancer was validated through CRISPR/Cas9 knockdown technology. Therefore, understanding the relationship between disease and miRNA can not only guide researchers to detect disease causality at the molecular level, but also promote the progress of human medicine and treatment of disease. Since traditional wet experiments are expensive, time-consuming and only work on tiny data. Consequently, there is a pressing requirement to develop efficient computational frameworks for detecting unobserved disease-associated miRNAs. Eventually, the proposed models for predicting disease-miRNA associations are roughly classified into five groups [9, 10]:

Methods based on complex scoring functions predict unknown associations by extracting disease-disease and miRNA-miRNA scoring terms. For instance, Jiang et al. [11] used a rating system to rank each predicted miRNAs according to the cumulative hypergeometric distribution of the disease and considered the top-ranked miRNAs as meaningful indicators. Later, Chen et al. [12] proposed WBSMDA to infer potential disease-miRNA relationships by combining existing associations with a range of similarities including gaussian interaction profile nuclear similarities.

Matrix-based methods predict unknown miRNA-disease associations by using various matrix completion or matrix factorization algorithms. For example, Chen et al. [13] constructed the IMCMDA algorithm to infer disease-miRNA correlations, which integrated disease similarity and miRNA similarity into an inductive completion matrix to obtain prediction scores. After that, Chen et al. [14] constructed a new algorithm NCMCMDA based on similarity information that merged similarity-based neighborhood constraints with matrix completion methods. Subsequently, MDHGI [15] employed the heterogeneous graph inference and the matrix factorization to detect disease-causing miRNAs. By combining the MISIM database [16] information with gaussian similarity, IMDN [17] created a miRNA similarity network and then performed matrix factorization of the association matrix with the regularized terms of the miRNA network. Zhu et al. [18] adopted the Bayesian Personalized Ranking algorithm to perform matrix decomposition for labeled interactions and fully utilized similarity information to enhance the accuracy of the prediction model. Recently, Wang et al. [19] designed the CKA-HGRTMF model to mine potential interactions between noncoding RNAs and diseases by introducing hypergraph and graph regularization terms.

Network propagation-based approaches predict potential disease-miRNA associations by using label propagation or graph inference algorithms. For example, Li et al. [20] iteratively propagated miRNA and disease label information into the constructed miRNA-disease network for association identification. Later, Chen et al. [21] constructed an algorithm called BNPMDA, which was a bipartite network projection algorithm based on known miRNA-disease correlations and bipartite graph network recommendation. However, BNPMDA was not suitable for disease prediction in the absence of any known relevant miRNA. Gong et al. [22] constructed a multi-information aggregation algorithm based on network embedding, called NEMII, which obtained the network features through the structural deep network embedding (SDNE) method and used the random forest algorithm for classification prediction. Specifically, the method revealed the superiority of random forest classifiers on unbalanced sample sets. Subsequently, Yu et al. [23] constructed an advanced model to detect correlations between diseases and miRNAs, which learned the potential representations of nodes by performing unbalanced random walks on a three-layer heterogeneous graph. In the case study, TCRWMDA was found to be a useful method for predicting disease-miRNA relationships. Moreover, MINIMDA [24] adopted the multilayer perceptron to discover the latent disease-associated miRNAs, which learned the feature representations of nodes from multimodal networks.

Machine learning-based methods excavate underlying disease-causing miRNAs based on regularization learning or recommendation algorithms. For example, Chen and Huang [25] proposed the LRSSLMDA method to reveal potential interactions, which utilized Laplacian regularization to learn local structure information from high dimensional spaces. To reduce the impact of noise in dataset, Liang et al. [26] constructed an adaptive learning-based approach to compute the correlation scores, which learned novel miRNA similarity graphs and disease similarity graphs from multiple views. Later, EDTMDA [27] utilized the principal components analysis to remove the redundant features and employed multiple decision trees to judge the interactions. As the unbalance samples would affect the prediction performance, ERMDA [28] applied the resampling algorithm to obtain several balanced training subsets and constructed individual learners to predict unlabeled associations.

Besides the calculation algorithms mentioned above, deep learning-based approaches predict miRNA-disease associations by propagating information from local neighbors with deep architectures such as graph convolutional network. Specifically, NIMCGCN [29] adopted graph convolutional network to extract characteristics and then fed them into an improved inductive matrix complementation algorithm. In addition, GCSENet [30] and PDMDA [31] constructed the full connection neural network and the softmax function to judge the correlations. Compared with GCSNet, PDMDA adopted three full connection layers to strengthen the ability of relationship prediction, which capitalized on the non-linear information. To fully exploit multiple views of the diseases and miRNAs, MMGCN [32] and MVGCN [33] employed multi-view graph convolutional architecture to make prediction. There are also several efforts to predict associations by using deep belief network and stacked autoencoder. In particular, considering that previous models only adopt known samples to train the network, Chen et al. [34] innovatively constructed a method named DBNMDA to mitigate the effect of the sparseness of validated miRNA-disease relationships on prediction, which learned the information of association pairs through a deep belief network during pre-training. Similar to DBNMDA, SAEMDA [35] first utilized both labeled samples and unlabeled samples to perform pre-training and fine-tuning by stacking three autoencoders and then excavated unobserved interactions based on the trained model. Further, Ji et al. [36] constructed AEMDA based on deep autoencoders, which employed the autoencoders for semi-supervised learning to predict unknown links. DFELMDA [37] introduced deep autoencoder to obtain low-dimensional embeddings and then applied deep random forest to estimate association probability. Meanwhile, with the popularity of graph attention mechanism in link prediction [38, 39], HGANMDA [40] designed semantic-layer and node-layer attention to weight different importance of meta-paths for excavating unobserved interactions.

Although the above algorithms have obtained great predictive capability, there are still some limitations for previous models as follows: first, approaches based on complex scoring functions are overly dependent on known miRNA-disease associations. Second, matrix-based approaches only capture linear associations, which are unable to accurately identify non-linear miRNA-disease interaction. Third, due to the lack of network structure information, network-based algorithms cannot acquire good performance in sparse networks. In addition, machine learning-based methods require feature engineering to improve the performance of the algorithm. Finally, current deep learning-based methods cannot effectively integrate multi-source data and use single-category features for prediction.

In order to solve the above problems, we propose a new computational model GATMDA, which effectively combines linear features and non-linear features based on multi-source data and graph attention networks to detect latent disease-miRNA relationships. Specifically, the whole process is summarized in the following three steps: first, we adopt lncRNA data as an intermediate node, which are combined with similarity data to obtain linear features of diseases and miRNAs respectively by matrix product algorithm. Second, we learn the miRNA-disease heterogeneous graph based on graph attention network to excavate the non-linear features of diseases and miRNAs. Third, the linear and non-linear features are cascaded to form new features of the node pairs, which are input into the random forest to get prediction scores. As a result, GATMDA achieves the prominent AUC of 0.9566 based on the benchmark dataset. Then case studies of breast cancer, colon cancer, and lymphoma could verify the model’s great independent predictive performance. In summary, GATMDA can significantly infer potential disease-miRNA relationships.

## Results

In this part, we deploy some validation experiments to assess the detective capability of GATMDA. First, we evaluate the effect of various parameter settings on GATMDA. Second, we design fivefold cross validation (CV) to assess the effect of GATMDA. Third, we compare and discuss GATMDA with state-of-the-art algorithms on miRNA datasets. Fourth, we further discuss the superiority of the graph attention mechanism over other feature processors in extracting features. Finally, case studies are designed to further verify the effectiveness in identifying candidate correlations on GATMDA.

### Parameter adjustment

**Fig. 1:**
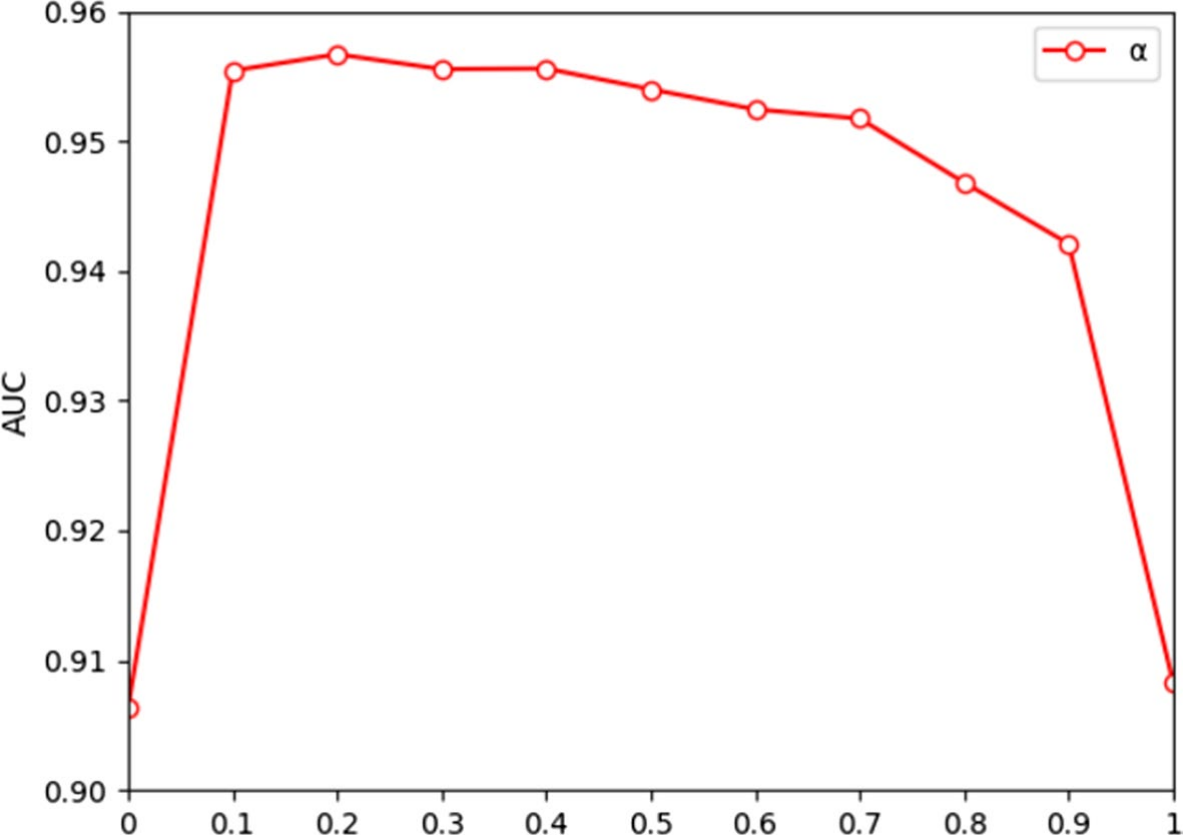
Comparison of the outputs for different dropout rate *α*

**Fig. 2:**
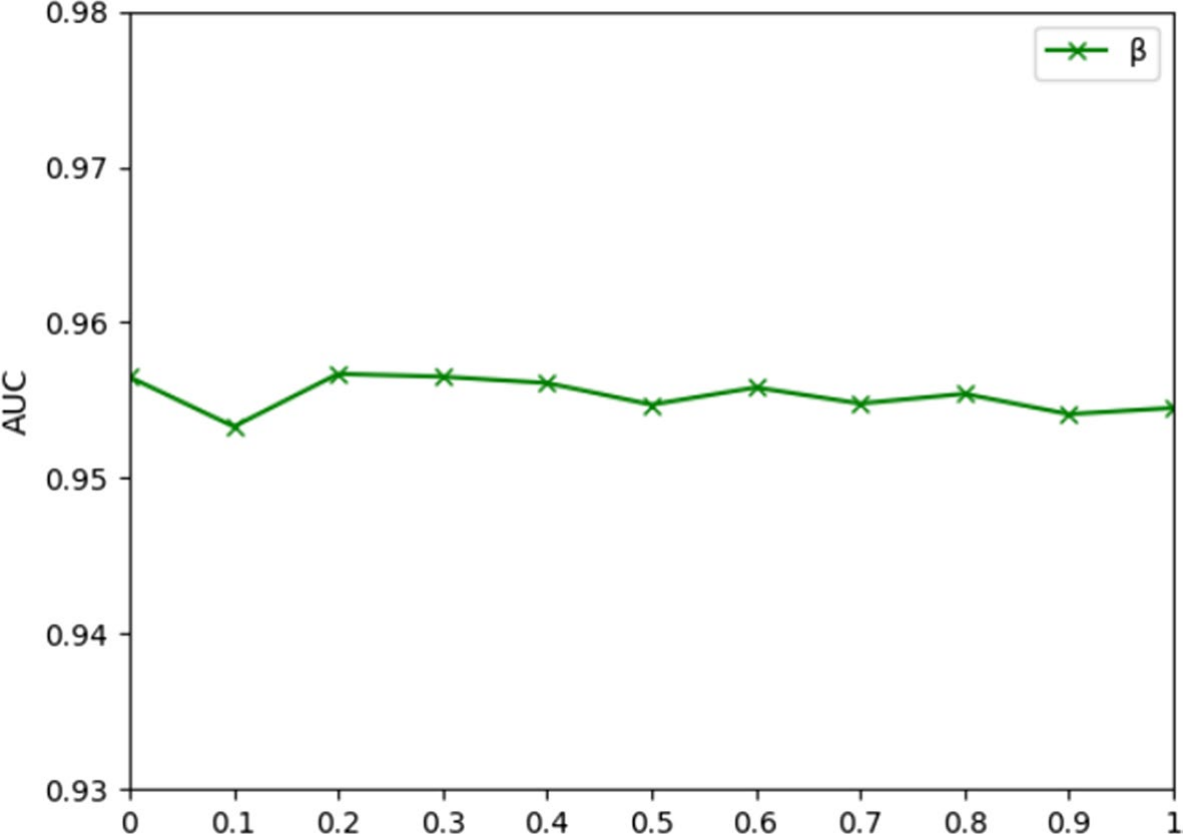
Comparison of the outputs for different activation parameters *β*

The predictive capability of an algorithm is usually affected by hyperparameter settings. It should be noted that we utilize fivefold CV to measure the effect of the parameters on the model performance. There are six parameters in the GATMDA method: *α*, *β*, *s*, *l’*, *r* and *λ*. First, *α* denotes the dropout rate, which is adopted to avoid GAT overfitting. We vary *α* from 0 to 1 with a step value of 0.1. As shown in Fig. 1, the performance of the model decreases as the rate of pruning rises, which indicates that the increase in the rate of pruning makes less information available for mining. The best performance is achieved when *α* = 0.2. We also vary the activation parameter *β* from 0 to 1 with a step value of 0.1. As *β* increases, the value of AUC does not change greatly. As shown in Fig. 2, the best result is achieved when *β* = 0.2. For the parameter *β,* which is used to avoid the vanishing gradients.

**Fig. 3:**
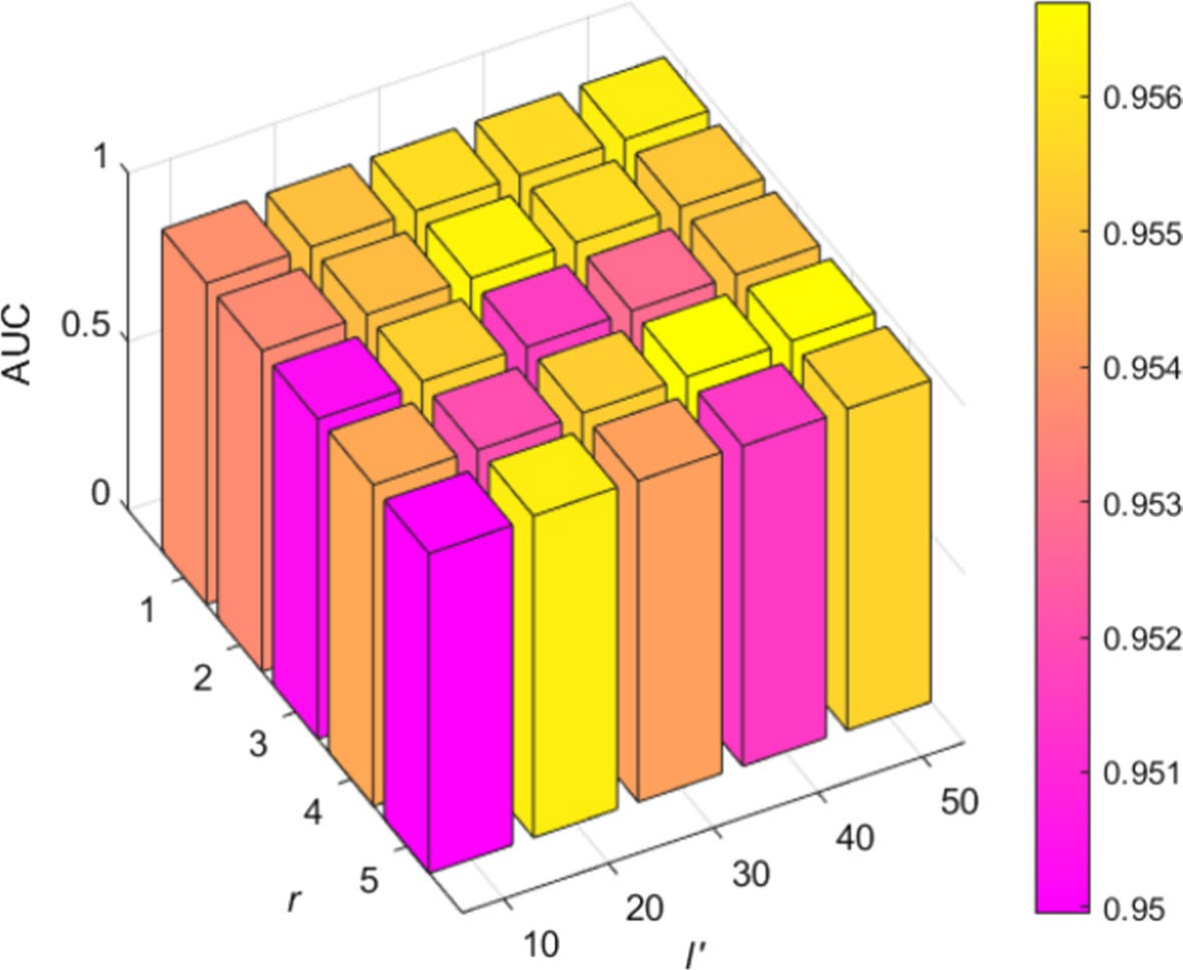
Effect of parameters *r* and *l’* in the results (*α* = 0.2, *β* = 0.2, *s* = 68)

For the number of neurons *s*, the best result of the model is obtained when we set *s* to 68. The dimension of non-linear feature *l’* plays an important role in our model. We range *l’* from 10 to 50 with a step value of 10. As shown in Fig. 3, low-dimensional non-linear features will lead to insufficient information extraction, while high-dimensional non-linear features will lead to redundancy information extraction. Later, we achieve the best result when *l’* = 40. *r* is the number of talking-heads. We vary *r* from 1 to 5 with a step value of 1. As shown in Fig. 3, we obtain the optimal AUC of the model at *r* = 4, which indicates that increasing the number of attention heads can mine more valid information.

**Fig. 4:**
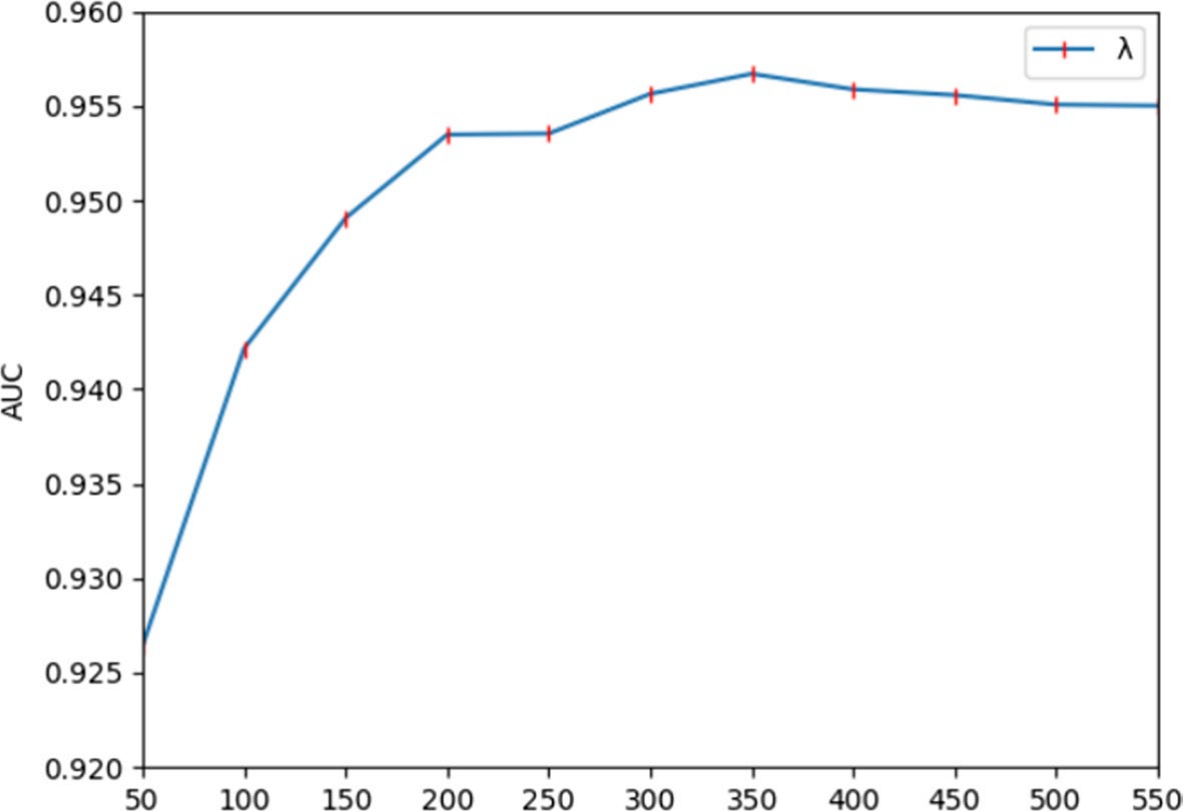
Comparing the output of different decision trees *λ*

Finally, after determining *α* = 0.2, *β* = 0.2, *s* = 68, *l’* = 40 and *r* = 4, the value of the decision tree *λ* is corrected. For the parameter *λ*, we set the range of values from 50 to 500 with a step size of 50. As shown in Fig. 4, we get the best performance when the random forest classifier has 350 trees.

### Performance evaluation

We measure the predictive performance of the model using a fivefold CV method, which randomly divides the positive samples into five subsets, one of which is used as the test sample and others as the training samples. The model repeats the fivefold CV 10 times to obtain the ultimate results which reduce the bias caused by sample segmentation. Subsequently, the predicted scores are ranked in descending order. We obtain the receiver operating characteristic curves (ROC) through drawing the false positive rate (FPR) with the true positive rate (TPR) at various scoring thresholds. The TPR (FPR) is the percentage of correctly identified positive (negative) cases. Typically, the area under the ROC curve (AUC) is computed and used to assess predictive capability of the model. In particular, when the AUC value is closer to 1, then the disease-miRNA relationship prediction performance is better. As a result, GATMDA obtains an AUC of 0.9566.

### Algorithm performance comparison

**Fig. 5:**
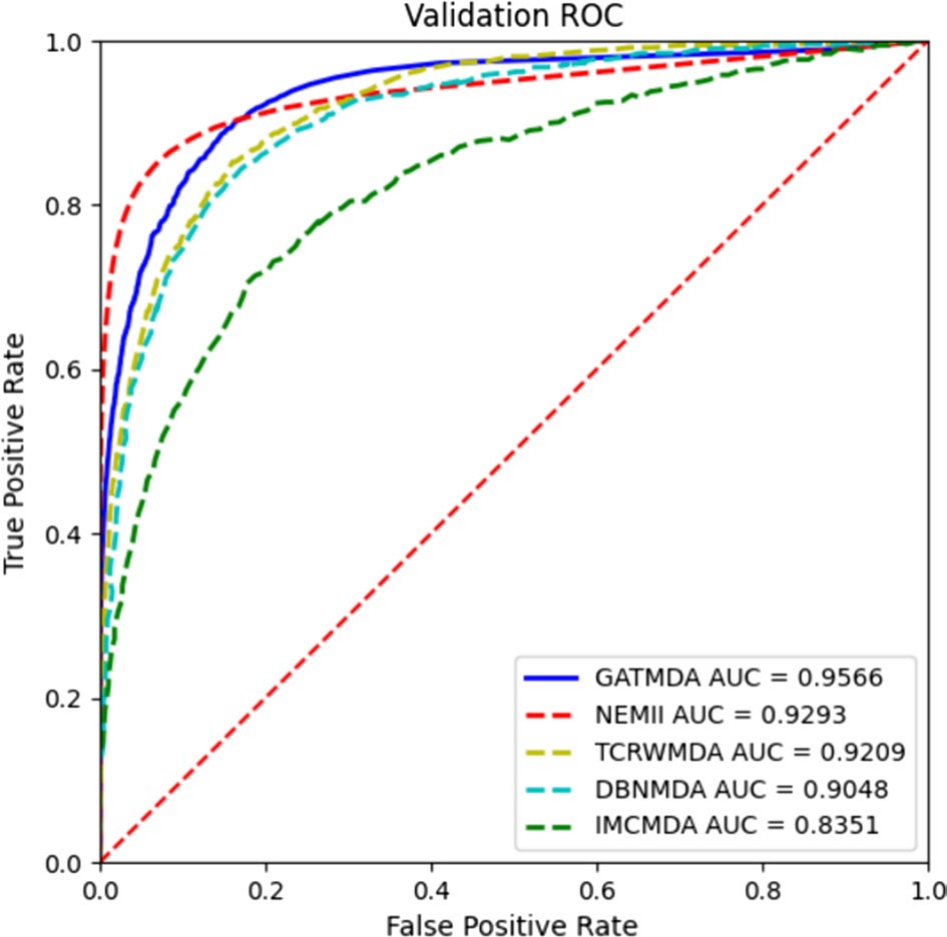
AUC values of GATMDA with other methods in the fivefold CV

To confirm the advantage of GATMDA in relationship prediction, we compare GATMDA with other state-of-the-art algorithms through 5-fold CV: IMCMDA [13], NEMII [22], TCRWMDA [23] and DBNMDA [34]. IMCMDA applied miRNA and disease similarity data as features to complement the disease-miRNA relationship matrix. NEMII used the structured deep network embedding method to obtain the nodes embedding in a bipartite network for predicting the relationships between diseases and miRNAs. TCRWMDA performed random walks on a three-layer heterogeneous network to obtain features for discovering disease-miRNA relationships. DBNMDA used deep belief networks to weaken the effect of limited known associations on prediction results. The prediction results of each method are displayed in Fig. 5. To be more specific, GATMDA produces the highest AUC score, obtaining average AUC of 0.9566, which has 2.73%, 3.57%, 5.18% and 12.15% higher than those of NEMII, TCRWMDA, DBNMDA and IMCMDA, respectively. As for the second highest NEMII model, it adopts first-order and second-order proximity to learn network structure, while our model exploits GAT to aggregate neighbors with different weights. Thus, NEMII fails to discriminate the importance among neighbors compared with GATMDA. TCRWMDA is based on random walk, which converts the network structure into sequence set. Thus, TCRWMDA fails to fully utilize the network structure information compared with GATMDA. IMCMDA is based on matrix complement, which is hard to model the non-linear relationships between diseases and miRNAs. Comparing with DBNMDA, GATMDA fuses linear and non-linear features. The possible reason is that linear features contain abundant shallow biological information and some noise, while non-linear features learned from disease-miRNA network can reduce the noise of linear feature and supplement deep structure information for linear features. Therefore, fusing linear and non-linear features can obtain comprehensive and complementary information for association prediction.

To further validate the superiority of GATMDA, we apply the paired *t*-test to measure whether there are statistical differences between GATMDA and other four methods. In Table 1, the statistical analysis suggests that there are very significant differences between GATMDA and compared models under the confidence level of 0.05. In conclusion, comparing with other models, GATMDA exhibits better results in fivefold CV.

**Table 1:** The differences between GATMDA and other models under fivefold CV

GATMDA vs	NEMII	TCRWMDA	DBNMDA	IMCMDA
*p*-value	5.8e−05	1e−04	2.6e−05	2.1e−06

### Ablation study

GATMDA is designed to predict latent links between diseases and miRNAs based on matrix multiplication method and graph attention network algorithm. To confirm the superiority of different components of GATMDA in prediction associations, we compare the results of GATMDA with four different feature processor combinations. First, we use the basic linear multiplication method to obtain linear features for prediction (combination 1). Second, we use the non-linear features obtained by GAT for prediction (combination 2). Third, linear features cascade the features extracted by the singular value decomposition (SVD) algorithm for prediction (combination 3). Finally, linear features cascade the features extracted by the DeepWalk algorithm for prediction (combination 4).

We use the AUC value of fivefold CV experiment to measure the effect of different feature combinations. In addition, we adopt several metrics to further assess the effectiveness of the GATMDA including accuracy (ACC), area under the precise-recall curve (AUPR), recall (REC), F1-measure (F1), specificity (SPEC) and precision (PRE).

According to the results in Table 2, the AUC value of GATMDA is significantly better than that of Combination 1 and Combination 2, which indicates that combining linear and non-linear features performs better than single-category feature for prediction. Then, the AUC value of combination 1 is lower than other combinations, indicating that association network features can supplement similarity features to improve the predictive capability of the model. Ultimately, the algorithm GATMDA outperforms combination 3 and combination 4, which shows that GAT feature processing is more suitable for disease-miRNA association prediction, since GAT is better at mining neighbor relationships.

**Table 2:** Model performance research based on different feature processing

Methods	ACC	F1	AUPR	REC	PRE	SPEC	AUC
Combination 1	0.992	0.335	0.463	0.358	0.352	0.995	0.906
Combination 2	0.995	0.510	0.468	0.412	0.675	0.998	0.923
Combination 3	0.996	0.601	0.459	0.485	0.794	0.999	0.937
Combination 4	0.996	0.597	0.477	0.483	0.788	0.999	0.939
GATMDA	0.996	0.606	0.477	0.498	0.781	0.999	0.956

### Comparison with other classifiers

**Fig. 6:**
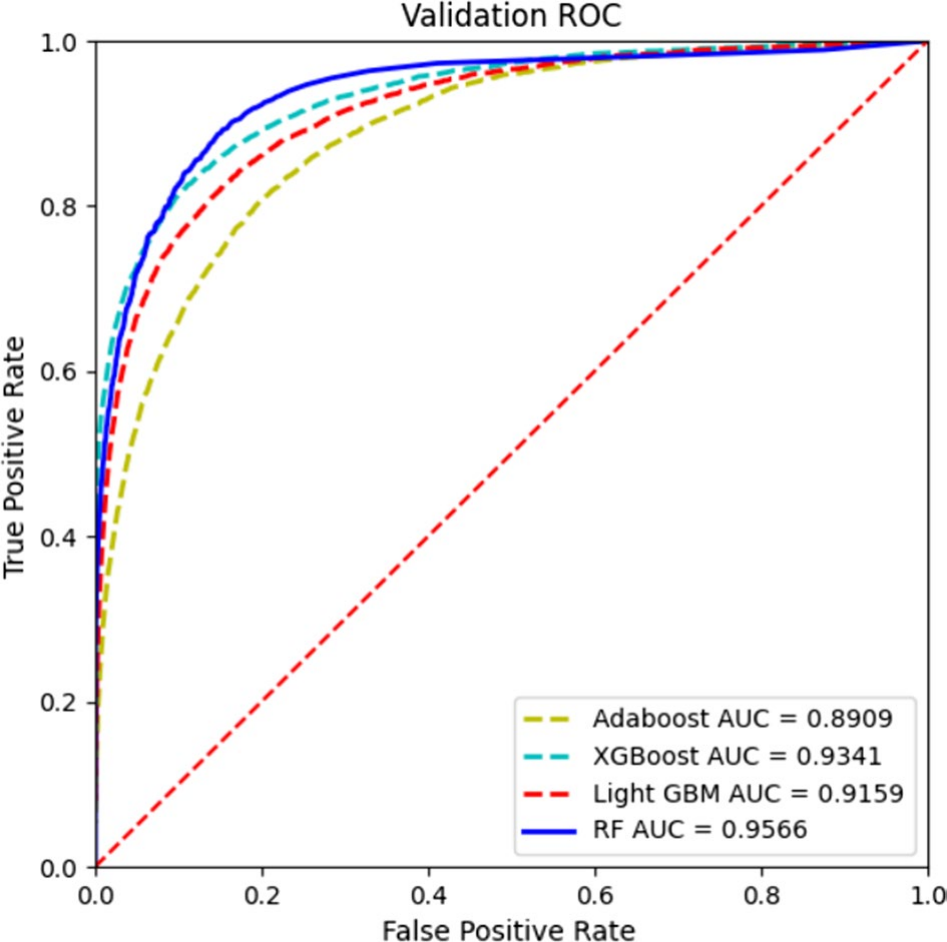
The ROC curves of GATMDA with other classifiers

GATMDA behaves well on HMDD v2.0 by utilizing the random forest (RF) algorithm. To prove that RF is the most suitable method for us, we compare the RF [41] algorithm with adaptive boosting (Adaboost), eXtreme gradient boosting (XGBoost) algorithm and Light gradient tree boosting machine (Light GBM). In the Adaboost algorithm, we set the learning rate to 0.7 and the resting parameter values to default. XGBoost classifier and Light GBM classifier all adopt default parameters. As show in Fig. 6, the AUC values of Adaboost, XGBoost, Light GBM and RF are 0.8909, 0.9341, 0.9159 and 0.9566, respectively. Simulation results prove that RF has higher AUC score than other models, because the RF algorithm is effective on high-dimensional datasets relative to the boosting algorithm [42].

### Robustness and significance validation

In order to evaluate the robustness of GATMDA, we further test the performance of GATMDA on another dataset named HMDD v3.2 [43]. We obtain the dataset of HMDD v3.2 from Li’s model [44], which includes 4189 interactions between 437 miRNAs and 431 diseases, 8172 relationships between 861 lncRNAs and 437 miRNAs, and 4518 lncRNA-disease correlations. To obtain a systematic and convincing comparison, we compare GATMDA method with several baselines on HMDD v3.2, including LAGCN [39], NEMII [22] and GCAEMDA [45]. LAGCN employed attention mechanisms to fuse the features of multiple graph convolutional layers for drug-disease association prediction. GCAEMDA constructed disease-based and miRNA-based subnetworks and adopted graph convolutional autoencoder to obtain association scores for the two subnetworks. Furthermore, the disease-miRNA prediction results of GCAEMDA were obtained by integrating the two association scores using an average ensemble approach.

**Fig. 7:**
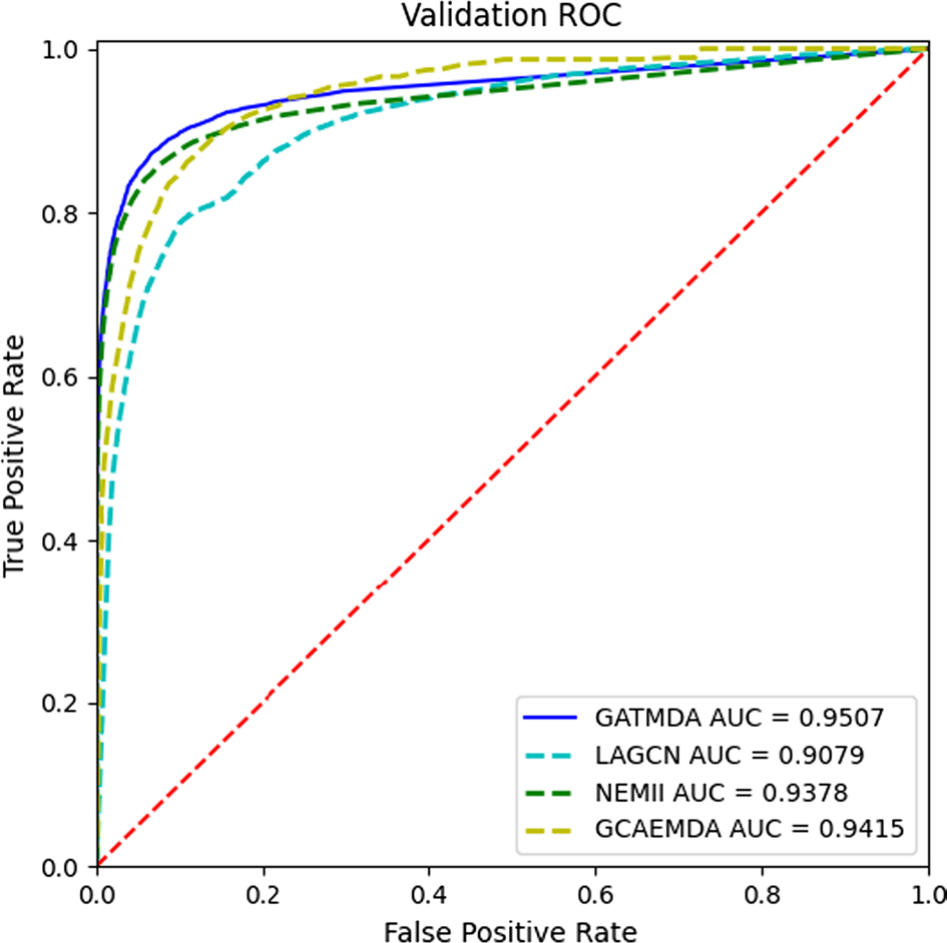
AUC values of GATMDA with other methods on HMDD v3.2

The comparison of the ROC curves obtained by different methods is shown in Fig. 7. It can be observed that GATMDA outperforms other compared models in terms of AUC under fivefold CV. The AUC scores of GATMDA, LAGCN, NEMII and GCAEMDA are 0.9507, 0.9079, 0.9385 and 0.9415, respectively. GATMDA achieves the best performance and makes 4.28%, 1.22% and 0.92% improvements in terms of AUC values, respectively. To further validate the superiority of GATMDA, we apply the paired *t*-test to measure whether there are statistical differences between GATMDA and other methods. In Table 3, the statistical analysis suggests that there are very significant differences between GATMDA and other three models under the confidence level of 0.05. These experimental results fully demonstrate the robustness and significance of GATMDA.

**Table 3:** The differences between GATMDA and other models under fivefold CV

GATMDA vs	LAGCN	NEMII	GCAEMDA
*p*-value	1.2e−04	5.8e−03	3.9e−03

**Fig. 8:**
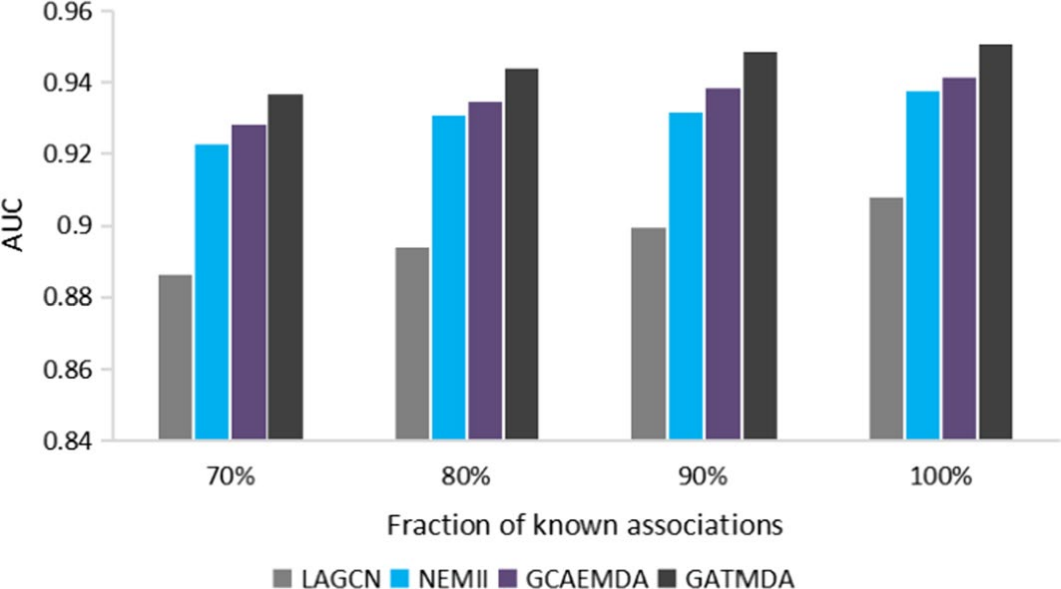
Performance of different predictors when removing associations

Moreover, we randomly remove a proportion of labeled interactions for further testing the scalability of GATMDA. As shown in Fig. 8, the AUC of GATMDA will decrease as removing more relationships, but it can still achieve AUC values higher than 0.92 when nearly 30% of the relationships are removed, which further proves the above conclusion about robustness of GATMDA.

### Prediction on multi-type associations

There are multiple categories of association pairs between disease and miRNA in HMDD. Therefore, the prediction of multi-category miRNA-disease interactions can not only reveal the specific interaction mechanism but also improve our understanding of how miRNAs cause disease. Currently, there are several efforts to study the issue of multi-category disease-miRNA interaction prediction. Chen et al. [46] conducted a pioneer study to excavate the specific association type by using restricted Boltzmann machine. By integrating the similarity of miRNA pairs and disease pairs, Zhang et al. [47] established a heterogeneous network and then applied label propagation to transfer each type of label information on the two integrated similarity networks. Afterwards, tensor decomposition algorithms are used to mine different types of disease-miRNA pairs, in which multi-category interactions are modeled as tensors. For instance, TDRC [48] employed tensor decomposition with relational constraints to establish the prediction model and TFLP [49] adopted label propagation in addition to tensor factorization. In order to obtain the multi-type and non-linear relationships between disease and miRNA, Wang et al. [50] leveraged the encoder and decoder of neural network to make prediction. Recently, Zhang et al. [51] introduced signed graph neural network model named SGNNMD to uncover the specific deregulation type. To investigate the performance of GATMDA for predicting multi-type disease-miRNA relationships, we implement GATMDA on a dataset annotated with two deregulation types. The used dataset is the same as that of SGNNMD [51], which contains 2284 up-regulation and 1980 down-regulation interactions between 348 miRNAs and 210 diseases. Here, we select SGNNMD [51] and two typical signed relationship prediction models (SNEA [52] and SGCN [53]) as baselines. As a result, the AUC values of GATMDA, SNEA, SGCN and SGNNMD are 0.749, 0.731, 0.617 and 0.917, respectively. Since GATMDA does not discriminate two types of edges when aggregating the features of nodes, it is not as effective as SGNNMD in classifying link categories, which adopts subgraphs to learn the difference between down-regulation and up-regulation interactions. In the future, we will exploit subgraph attention mechanisms to enhance the feature learning ability of the model for excavating specific regulation type.

### Case studies

To further assess the effectiveness of GATMDA in inferring latent disease-miRNA relationships, we predict the probability matrix based on all known association set. Then, the probability matrix is sorted by score descending to select disease-related top 50 candidate miRNAs. Since all known relationships in the dataset are utilized to train the model, the predicted candidates need to be validated by known databases and literature, such as dbDEMC [54], HMDD v3.2 [43] and PubMed. Finally, we conduct case studies of three common diseases, including colon cancer, breast cancer and lymphoma.

Breast cancer is the major cancer in women and the main cause of cancer death around the world. Table 4 indicates that all relationships in the top 50 detected breast cancer candidate miRNAs are experimentally validated. For example, ectopic expression of miR-150 inhibited migration of TNBC cells and metastasis, which downregulated in TNBC tumor tissue compared to adjacent normal breast tissue [55]. Has-miR-106a regulated heat shock proteins to affect human breast cancer [56].

**Table 4:** Top 50 candidate miRNAs predicted by GATMDA to be connected with breast cancer

miRNA (1–25)	Evidence	miRNA (26–50)	Evidence
hsa-mir-198	HMDD; dbDEMC	hsa-mir-566	dbDEMC
hsa-mir-150	dbDEMC	hsa-mir-582	dbDEMC
hsa-mir-208b	dbDEMC	hsa-mir-589	dbDEMC
hsa-mir-106a	HMDD; dbDEMC	hsa-mir-619	dbDEMC
hsa-mir-192	HMDD; dbDEMC	hsa-mir-627	dbDEMC
hsa-mir-449a	HMDD; dbDEMC	hsa-mir-635	dbDEMC
hsa-mir-449b	dbDEMC	hsa-mir-650	dbDEMC
hsa-mir-95	dbDEMC	hsa-mir-655	dbDEMC
hsa-mir-99b	dbDEMC	hsa-mir-744	dbDEMC
hsa-mir-1180	dbDEMC	hsa-mir-942	dbDEMC
hsa-mir-1184	dbDEMC	hsa-mir-484	dbDEMC
hsa-mir-1246	dbDEMC	hsa-mir-503	dbDEMC
hsa-mir-1247	dbDEMC	hsa-mir-99a	HMDD; dbDEMC
hsa-mir-1273a	dbDEMC	hsa-mir-130a	HMDD; dbDEMC
hsa-mir-1302	dbDEMC	hsa-mir-382	dbDEMC
hsa-mir-196b	dbDEMC	hsa-mir-483	dbDEMC
hsa-mir-1972	dbDEMC	hsa-mir-15b	dbDEMC
hsa-mir-33a	dbDEMC	hsa-mir-28	dbDEMC
hsa-mir-362	dbDEMC	hsa-mir-376a	dbDEMC
hsa-mir-374b	dbDEMC	hsa-mir-424	dbDEMC
hsa-mir-378a	HMDD; dbDEMC	hsa-mir-491	HMDD; dbDEMC
hsa-mir-421	dbDEMC	hsa-mir-675	HMDD; dbDEMC
hsa-mir-433	dbDEMC	hsa-mir-410	HMDD; dbDEMC
hsa-mir-454	dbDEMC	hsa-mir-144	dbDEMC
hsa-mir-519a	dbDEMC	hsa-mir-181c	HMDD; dbDEMC

Colon cancer is a frequent malignant neoplasm of the digestive system that develops in the colon. The results in Table 5 show that all associations in the top 50 detected colon cancer candidate miRNAs are experimentally confirmed. For instance, Yan et al. demonstrated that hsa-miR-125a was upregulated in human colon cancer cells (SW480) [57]. Wang et al. [58] discovered that miR-29a inhibited the evolution of colon cancer by down-regulating the B7-H3 expression.

**Table 5:** Top 50 candidate miRNAs predicted by GATMDA to be connected with colon cancer

miRNA (1–25)	Evidence	miRNA (26–50)	Evidence
hsa-mir-125a	HMDD; dbDEMC	hsa-mir-191	dbDEMC
hsa-mir-196a	dbDEMC	hsa-mir-192	HMDD; dbDEMC
hsa-mir-499a	dbDEMC	hsa-mir-193b	dbDEMC
hsa-mir-198	dbDEMC	hsa-mir-194	dbDEMC
hsa-mir-29a	dbDEMC	hsa-mir-195	HMDD; dbDEMC
hsa-mir-29b	dbDEMC	hsa-mir-200a	dbDEMC
hsa-let-7a	HMDD; dbDEMC	hsa-mir-200b	dbDEMC
hsa-mir-141	dbDEMC	hsa-mir-200c	HMDD; dbDEMC
hsa-mir-143	HMDD; dbDEMC	hsa-mir-203	dbDEMC
hsa-mir-150	dbDEMC	hsa-mir-204	dbDEMC
hsa-mir-15a	dbDEMC	hsa-mir-205	HMDD; dbDEMC
hsa-mir-16	dbDEMC	hsa-mir-20a	HMDD; dbDEMC
hsa-mir-21	HMDD; dbDEMC	hsa-mir-210	dbDEMC
hsa-mir-1	HMDD; dbDEMC	hsa-mir-215	HMDD; dbDEMC
hsa-mir-133a	HMDD; dbDEMC	hsa-mir-221	HMDD; dbDEMC
hsa-mir-133b	dbDEMC	hsa-mir-223	dbDEMC
hsa-mir-146a	dbDEMC	hsa-mir-25	dbDEMC
hsa-mir-155	HMDD; dbDEMC	hsa-mir-26b	dbDEMC
hsa-mir-103a	dbDEMC	hsa-mir-31	HMDD; dbDEMC
hsa-mir-10b	HMDD; dbDEMC	hsa-mir-34b	dbDEMC
hsa-mir-135a	dbDEMC	hsa-mir-429	dbDEMC
hsa-mir-151a	dbDEMC	hsa-mir-449b	dbDEMC
hsa-mir-181b	dbDEMC	hsa-mir-92a	HMDD; dbDEMC
hsa-mir-182	dbDEMC	hsa-mir-93	dbDEMC
hsa-mir-183	dbDEMC	hsa-mir-95	dbDEMC

Malignant lymphomas represent a range of different diseases that arise from the clonal proliferation of lymphocytes. Table 6 lists the top 50 candidate miRNAs from the predicted results, of which 48 associations are experimentally validated. For example, over-expression of mir-196a was inhibition of multiplication in a non-Hodgkin's lymphoma and enhancing apoptosis [59]. Cécile et al. [60] demonstrated miR-29a as a potential tool to influence lymphoma tumorigenesis.

**Table 6:** Top 50 candidate miRNAs predicted by GATMDA to be connected with lymphoma

miRNA (1–25)	Evidence	miRNA (26–50)	Evidence
hsa-mir-196a	dbDEMC	hsa-mir-205	dbDEMC
hsa-mir-198	dbDEMC	hsa-mir-215	dbDEMC
hsa-mir-29a	dbDEMC	hsa-mir-221	HMDD; dbDEMC
hsa-mir-29b	dbDEMC	hsa-mir-223	dbDEMC
hsa-let-7a	dbDEMC	hsa-mir-25	dbDEMC
hsa-mir-141	dbDEMC	hsa-mir-26b	dbDEMC
hsa-mir-143	HMDD; dbDEMC	hsa-mir-31	HMDD; dbDEMC
hsa-mir-145	dbDEMC	hsa-mir-34b	dbDEMC
hsa-mir-1	dbDEMC	hsa-mir-429	PMID34651663
hsa-mir-133a	dbDEMC	hsa-mir-449a	dbDEMC
hsa-mir-208b	dbDEMC	hsa-mir-449b	dbDEMC
hsa-mir-103a	dbDEMC	hsa-mir-93	HMDD; dbDEMC
hsa-mir-106a	dbDEMC	hsa-mir-95	dbDEMC
hsa-mir-10b	HMDD; dbDEMC	hsa-mir-99b	dbDEMC
hsa-mir-151a	dbDEMC	hsa-let-7e	dbDEMC
hsa-mir-152	dbDEMC	hsa-mir-1180	dbDEMC
hsa-mir-181b	dbDEMC	hsa-mir-1184	unconfirmed
hsa-mir-182	dbDEMC	hsa-mir-1246	dbDEMC
hsa-mir-183	dbDEMC	hsa-mir-1247	dbDEMC
hsa-mir-191	dbDEMC	hsa-mir-125b	dbDEMC
hsa-mir-192	dbDEMC	hsa-mir-1273a	unconfirmed
hsa-mir-193b	HMDD; dbDEMC	hsa-mir-1302	dbDEMC
hsa-mir-194	HMDD; dbDEMC	hsa-mir-146b	dbDEMC
hsa-mir-195	dbDEMC	hsa-mir-148a	dbDEMC
hsa-mir-204	HMDD; dbDEMC	hsa-mir-148b	dbDEMC

The case study results indicate that GATMDA can effectively detect latent disease-miRNA associations, which provides ideas for discovering the mechanisms between miRNAs and complex human diseases.

### Differential expression analysis and survival analysis

**Fig. 9:**
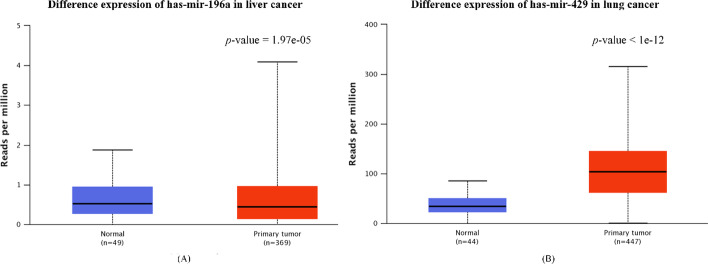
**A** Differential expression analysis of has-mir-196a in liver cancer; **B** differential expression analysis of has-mir-429 in lung cancer

To verify whether some top predicted miRNAs can be confirmed by biological experiments, we perform the differential expression analysis and Kaplan–Meier survival analysis using the clinical data and expression value obtained from The Cancer Genome Atlas (TCGA). Specifically, we select the top predicted miRNA has-mir-196a (first in the prediction list) in liver cancer and has-mir-429 (first in the prediction list) in lung cancer for analysis respectively. The results of differential expression analysis are displayed in Fig. 9. We can observe that the expression level of these two selected miRNAs in tumor group comparting with normal group is significantly altered.

**Fig. 10:**
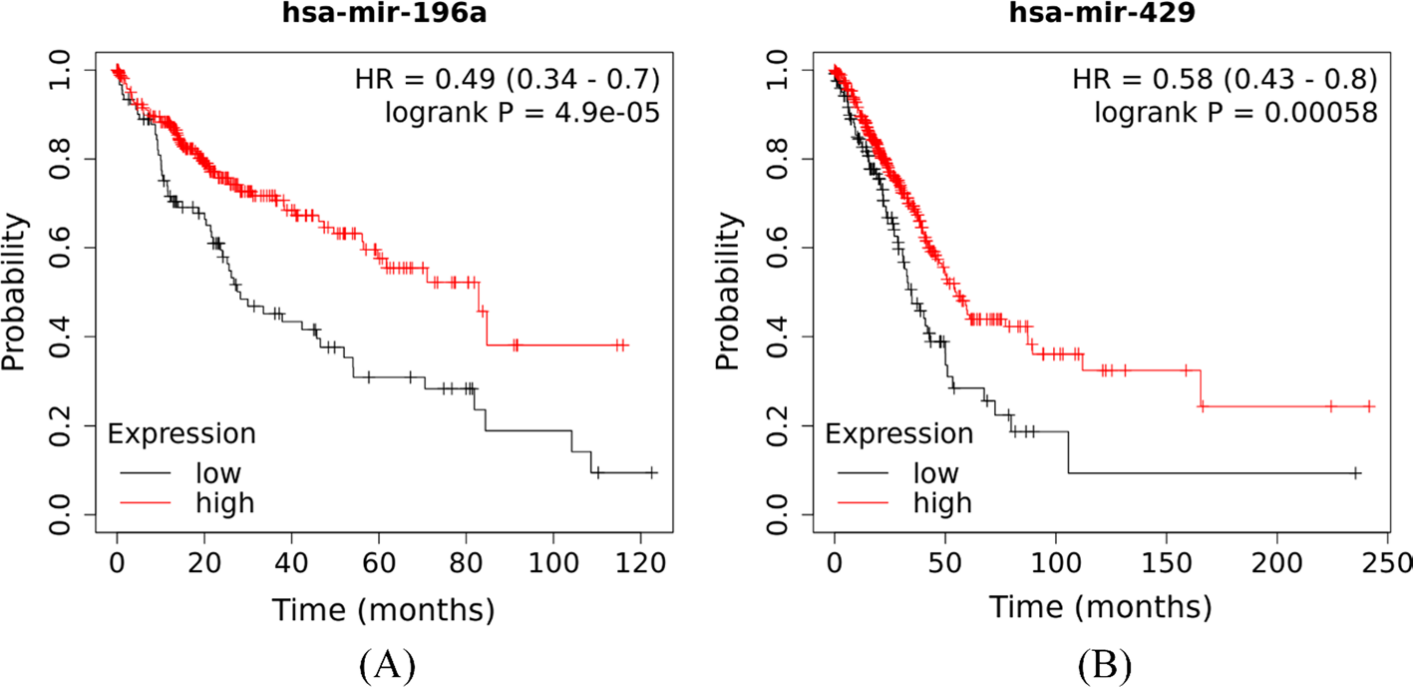
**A** Survival analysis of has-mir-196a in liver cancer; **B** survival analysis of has-mir-429 for in lung cancer

The results of survival analysis are displayed in Fig. 10. We can see that these two selected miRNAs are significantly related to the survival rates of patients. In particular, the liver cancer and lung cancer patients with higher expression values of has-mir-196a and has-mir-429 respectively will both have a higher survival rate. These results suggest that high-ranked miRNAs predicted by GATMDA may play key role in early diagnosis and prognosis of tumors.

## Discussion

Experimental results compared with four association prediction models in fivefold CV demonstrate that GATMDA outperforms other prediction models. We analyze the impact of different feature processors and different feature combinations. In addition, case studies conducted on three diseases justify the predictive performance of our model. The success of GATMDA is attributed from three factors. First, we strengthen linear feature mining of miRNAs and diseases through miRNA-lncRNA correlation profile and disease-lncRNA correlation profile. Second, we employ the graph attention network to extract non-linear features of miRNAs and diseases by aggregating information with different weights for each neighbor. Third, non-linear features are used to supplement linear features for association prediction. In summary, GATMDA achieves excellent prediction performance by effectively fusing linear and non-linear representations in miRNA-disease association network. However, our work still has several limitations that are expected to be further enhanced in the future. On the one hand, due to the sparseness of disease-lncRNA correlation profiles and miRNA-lncRNA correlation profiles, the feature information provided by multi-source data is limited. In the future, we will collect more lncRNA association data to enhance the quality of linear features. On the other hand, the dimensionality of linear features may be high if more lncRNA data are introduced. To solve this problem, PCA method is used to reduce the dimension of linear features and reduce the influence of noise.

## Conclusion

Identifying new disease-miRNA relationships is significant for exploring the pathogenesis of diseases and improving human medicine. However, previous computational algorithms suffer from two main challenges. On the one hand, the proven miRNA-disease associations are rare, resulting in limited information that can be extracted. On the other hand, the type of feature is limited, and the complex relationships between miRNAs and diseases are difficult to express with single-category feature. Thus, we construct a new deep learning computational model, named GATMDA, based on graph attention network with multi-source data to identify potential disease-miRNA relationships. First, based on the lncRNA-miRNA-disease regulatory mechanism, we use lncRNA as multi-source biological information to enhance node linear feature expression. Second, we process the miRNA-disease graph using GAT to extract node non-linear features. In particularly, comparative experiments reveal that GAT assigns different learning weights to different neighbors, which can strengthen the mining of neighbor information between nodes. Finally, we combine the initial linear features with the depth non-linear features to form node new features for association prediction. To prove the advantages of GATMDA in predicting disease-miRNA correlations, we compare GATMDA with four detection models in fivefold CV. The results confirm that GATMDA performs better than the other detection models. Furthermore, case studies demonstrate that our model can effectively detect latent disease-miRNA relationships. In summary, GATMDA is a powerful framework for excavating disease-miRNA links. In the future, we will use alternative feature learning methods (such as variational graph auto-encoder algorithm) to strengthen node similarity features.

## Methods

### MiRNA-disease association dataset

The disease-miRNA relationship dataset is derived from HMDD v2.0 [61], which contains 5430 known biologically validated relationships between 383 diseases and 495 miRNAs. We adopt the adjacency matrix *MD* to denote the known relationship between disease *d*(*j*) and miRNA *m*(*i*). If *MD*(*i*, *j*) = 1, it means that disease *d*(*j*) is connected with miRNA *m*(*i*), otherwise, *MD*(*i*, *j*) = 0.

### MiRNA-lncRNA association dataset

From Star-base v2.0 [62], we collect 704 experimentally confirmed correlations between 495 miRNAs and 34 lncRNAs. The miRNA-lncRNA relationships are denoted as the adjacency matrix *ML*. If *ML*(*i*, *j*) = 1, it represents that lncRNA *l*(*j*) is connected with miRNA *m*(*i*), otherwise, *ML*(*i*, *j*) = 0.

### Disease-lncRNA association dataset

We download 182 experimentally confirmed correlations between 383 diseases and 34 lncRNAs from the dataset which are published by Chen et al. [63]. *DL* represent the disease-lncRNA connection matrix, *DL*(*i*, *j*) = 1 indicates that disease *d*(*i*) is correlated with lncRNA *l*(*j*), otherwise *DL*(*i*, *j*) = 0.

### MiRNA functional similarity

Wang et al. [16] established an algorithm for computing miRNA functional similarity, which is on the basis of the notion that similar miRNAs are usually to be relevant with similar diseases. We are benefiting from Wang’s research and download the miRNA similarity at http://www.cuilab.cn. The similarity scores between miRNAs *m*(*i*) and *m*(*j*) are represented by *FS*(*m*(*i*), *m*(*j*)).

### Disease semantic similarity

Semantic similarity model 1: The relationships among different diseases can be downloaded from MeSH descriptor [64], which use Directed Acyclic Graph (DAG) to represent them. The semantic contribution of disease *d* to disease *D* can be expressed by Eq. (1):

1 $$\left\{ {\begin{array}{*{20}l} {D1_{D} \left( d \right) = 1} \hfill & {if\quad d = D} \hfill \\ {D1_{D} \left( d \right) = \left\{ {\Delta *D1_{D} \left( {d^{\prime}} \right)\hfill| \hfill d^{\prime} \in \;children\;of\;d} \right\}} \hfill & {if\quad d \ne D} \hfill \\ \end{array} } \right.$$

△ represents the decay factors of semantic contribution. Therefore, the semantic value of disease *D* can be denoted by Eq. (2):

2 $$\begin{array}{*{20}c} {DV1\left( {\text{D}} \right) = \sum\limits_{d \in T\left( D \right)} {D1_{D} \left( d \right)} } \\ \end{array}$$

where *T*(*D*) denotes the ancestor nodes and *D* itself. Based on the hypothesis that if diseases *d*(*i*) and *d*(*j*) have a high portion of similarity in DAG, then these two diseases are more similar. The semantic similarity of disease *d*(*j*) and *d*(*i*) is expressed by Eq. (3):

3 $$\begin{array}{*{20}c} {{\text{DS}} 1\left( {d(i),d(j)} \right) = \frac{{\mathop \sum \nolimits_{{d \in T\left( {d{(}i{)}} \right) \cap T\left( {d{(}j{)}} \right)}} \left( {D1_{{d{(}i{)}}} \left( d \right) + D1_{{d{(}j{)}}} \left( d \right)} \right)}}{{{\text{DV}} 1\left( {d{(}i{)}} \right) + {\text{DV}} 1\left( {d{(}j{)}} \right)}}} \\ \end{array}$$

Semantic similarity model 2: Since the frequency of disease occurrence in the same layer of DAG might be distinct, we further integrate the algorithm constructed by Xuan et al. [65] to compute semantic similarity. The contribution to disease *d* in DAG(*d*) can be written by Eq. (4):

4 $$\begin{array}{*{20}c} {D2_{D} \left( d \right) = - \log \left[ {\frac{{ {\text{the}} \;number\;of\;DAGs\;{\text{including}} \;d}}{{ {\text{the}} \;number\;of\;diseases}}} \right]} \\ \end{array}$$

Then, we calculate semantic similarity *DS*2 of disease *d*(*j*) and *d*(*i*) as the ratio of their common ancestor node’s contribution to their own contributions as follows:

5 $${\text{DS}} 2{(}d{(}i{)},d{(}j{))} = \frac{{\sum\nolimits_{{d \in T_{{d{(}i{)}}} \cap T_{{d{(}j{)}}} }} {{(}D2_{{d{(}i{)}}} {(}d{)} + D2_{{d{(}j{)}}} {(}d{))}} }}{{{\text{DV}} 2{(}d{(}i{))} + {\text{DV}} 2{(}d{(}j{))}}}$$

Among them, the semantic value of disease *D* is calculated by Eq. (6):

6 $$\begin{array}{*{20}c} {{\text{DV}} 2\left( {\text{D}} \right) = \sum\limits_{d \in T\left( D \right)} {D2_{D} \left( d \right)} } \\ \end{array}$$

Finally, we take the average of *DS*1 and *DS*2 as the disease similarity *D*_*s*_.

### GATMDA

**Fig. 11:**
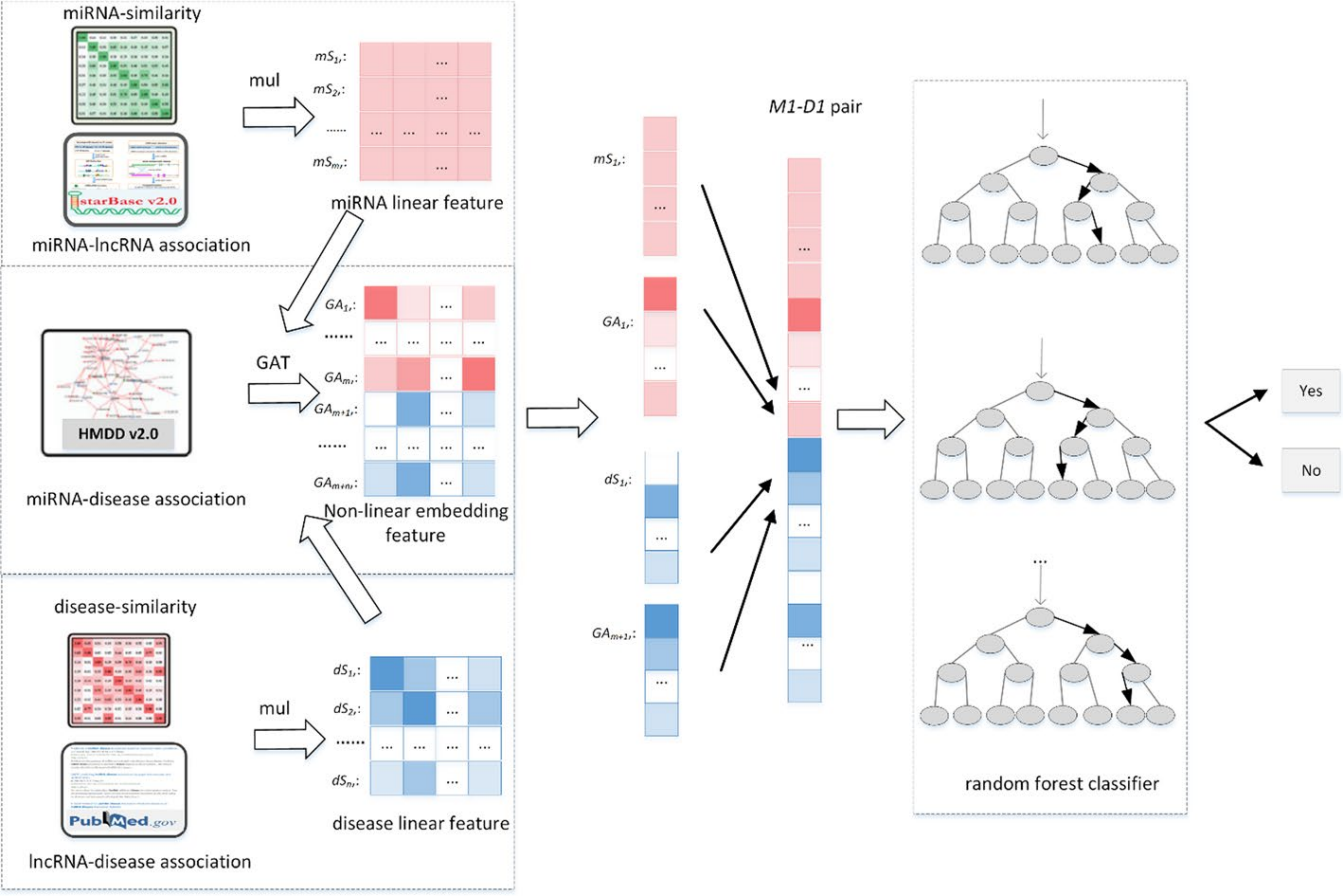
Flow chart of the calculation method GATMDA

In this work, we construct an advanced algorithm GATMDA through graph attention networks (GAT) with multi-source data to infer latent disease-miRNA connections. As shown in Fig. 11, GATMDA can be summarized in the following four steps: first, we use the linear multiplication method to incorporate lncRNA association data with similarity data for obtaining the linear features of miRNAs and diseases. Second, we construct GAT to learn the deep representation in miRNA-disease heterogeneous graph to obtain the non-linear features of nodes. Third, we use cascade operation to fuse linear and non-linear features into new features for miRNA-disease pairs. Finally, we employ a random forest algorithm as a categorization engine to classify disease-miRNA pairs.

### Constructing node linear features

Since experimentally confirmed miRNA-disease associations are limited, we adopt multi-source data to solve the association data sparsity problem. Considering that both lncRNAs and miRNAs are critical regulators which influence cellular activities and cause some diseases by regulating gene expression [66], we use lncRNA correlation data to enhance disease and miRNA feature information. To retain more initial information of node similarity, we use the linear multiplication method to extract the linear features from the similarity network and lncRNA correlation data. Specifically, the miRNA linear features are generated by multiplying the miRNA functional similarity *FS* with the miRNA-lncRNA correlation profiles *ML*.

7 $$\begin{array}{*{20}c} {F_{m} = FS \times ML} \\ \end{array}$$

Similarly, we perform linear multiplication method on the disease semantic similarity matrix *D*_*s*_ and the disease-lncRNA correlation profiles *DL* to obtain the disease linear features.

8 $$\begin{array}{*{20}c} {F_{d} = D_{s} \times DL} \\ \end{array}$$

We assume that there are *m* miRNAs, *n* diseases and *l* lncRNAs. Subsequently, each disease and miRNA can be represented by an *l* dimensional vector. Eventually, we use *F* to represent the features of all diseases and miRNAs as follows:

9 $$\begin{array}{*{20}c} {F = \left[ {\begin{array}{*{20}c} {\begin{array}{*{20}c} {F_{m} } \\ {F_{d} } \\ \end{array} } \\ \end{array} } \right]^{ } = \left[ {\begin{array}{*{20}c} {f_{1} } \\ {f_{2} } \\ \cdots \\ {f_{m + n} } \\ \end{array} } \right] \in R^{{\left( {m + n} \right) \times l}} } \\ \end{array}$$

where *m* + *n* represents the overall number of nodes, and *f* ∈ *R*^*l*^ denotes the linear feature of each node.

### Constructing graph attention non-linear features

Since the relationships between diseases and miRNAs are very complex, using pure linear feature is insufficient to mine potential information between miRNAs and diseases. To solve this problem, we use graph attention network [67] in the miRNA-disease graph to learn the non-linear features of diseases and miRNAs respectively. In particular, GAT first implements a self-attention mechanism for a given node to calculate the importance of its neighbors, and subsequently the given node feature is updated by aggregating the features of all the neighbors according to their attention coefficients. In this section, we fist construct the disease-miRNA graph based on interaction matrix *MD*, and define it as *G* = (*V*, *E*). *V* = {*v*_1_, *v*_2_, …, *v*_*m*_ + *v*_*n*_} are vertices, *E* represents the edges between miRNAs and diseases, and *F* are initial features of nodes in graph *G*. Then, we apply attention mechanism to learn the importance of a given node and its neighbor. Specifically, the attention coefficient *e*_*ij*_ between node *n*_*i*_ and its neighbor *n*_*j*_ is calculated as follows:

10 $$\begin{array}{*{20}c} {e_{ij} \left( {n_{i} ,n_{j} } \right)} \\ \end{array} = leaky{\text{Re}} Lu\left( {a^{T} \left[ {Wf_{i} ||Wf_{j} } \right]} \right)$$

where *W* ∈ *R*^*l'*×*l*^ denotes a transformation matrix to project the initial node feature into the *l’*-dimensional space, and *leakyReLu* denotes a non-linear activation function that assigns a non-zero slope to all negative values. *a* ∈ *R*^2*l'*^ denotes the attention parameter, which maps features to a real number.

Subsequently, we further normalize the attention coefficients *e*_*ij*_ to eliminate the dimensional influence between different attention coefficients as follows:

11 $$\begin{array}{*{20}c} {\theta_{ij} = {\text{softmax}} \left( {e_{ij} } \right) = \frac{{{\text{exp}}\left( {e_{ij} } \right)}}{{\mathop \sum \nolimits_{{t \in N_{i} }} {\text{exp}}\left( {e_{it} } \right)}}} \\ \end{array}$$

where *N*_*i*_ denotes the group of neighbor nodes of node *n*_*i*_. *θ*_*ij*_ represents the normalized attention coefficient indicating the importance between node *n*_*j*_ and *n*_*i*_.

Eventually, we use these attention coefficients to update the representations of the given node *n*_*i*_ by aggregating information from its neighbors:

12 $$f_{i}^{^{\prime}} \user2{ = }\sigma \left( {\sum\limits_{{t \in N_{i} }} {\theta_{it} } Wf_{t} } \right)$$

where *σ* is the LeakyReLU activation function.

To stabilize the result and reduce the bias, we use multi-head attention to steady the learning results of self-attention and strengthen the information extraction ability of our model. Since each head picks information from different representation spaces, multi-head attention can efficiently enhance information capture capability based on different learning focus. Specifically, integrating the *K*-independent attention mechanism to obtain vectors is as follows:

13 $$f_{i} \user2{^{\prime}} = \sigma \left( {\frac{1}{K}\sum\limits_{k = 1}^{K} {\sum\limits_{{t \in N_{i} }} {\theta_{it}^{k} \cdot W^{k} f_{t} } } } \right)$$

where *K* denotes the number of attention mechanisms.

Finally, the output of the graph attention layer is:

14 $$\begin{array}{*{20}c} {\begin{array}{*{20}c} {F^{\prime} = \left[ {\begin{array}{*{20}c} {f^{\prime}_{1} } \\ {f^{\prime}_{2} } \\ \cdots \\ {f^{\prime}_{m + n} } \\ \end{array} } \right]} \\ \end{array} \in R^{{\left( {m + n} \right) \times l^{\prime}}} } \\ \end{array}$$

where *l′* represents the dimension of new features, matrix *F′* ∈ *R*^*(m*+*n)*×*l′*^ denotes the learned potential representation of all nodes in the network. We use matrix *F*_*m*_*′* ∈ *R*^*m*×*l′*^ to represent the new features of all miRNA nodes. Similarly, *F*_*d*_*′* ∈ *R*^*n*×*l′*^ represents the new features of diseases nodes.

**Fig. 12:**
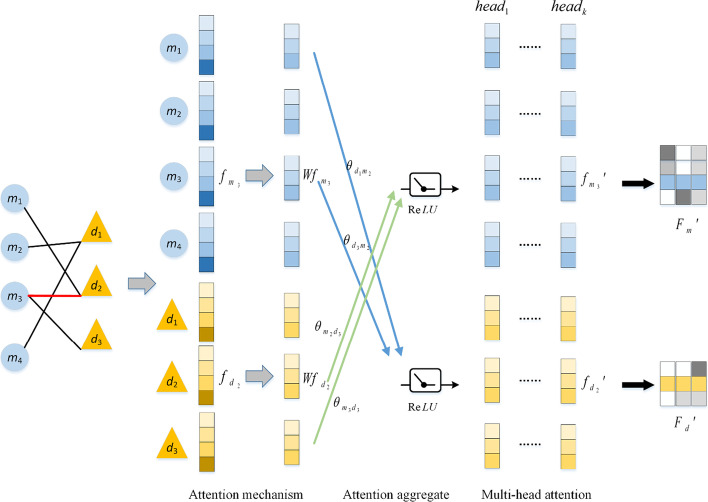
Detailed steps for obtaining non-linear embeddings of miRNA and disease using GAT

The detailed steps of using GAT to obtain disease non-linear feature vector *F*_*d*_*′* and miRNA non-linear feature vector *F*_*m*_*′* are displayed in Fig. 12. The miRNA-disease association graph and the linear features *F* possessed by each node are fed into GAT. Eventually, non-linear node representation is obtained through feature propagation and attention fusion.

### Fusion of linear and non-linear features to represent miRNA-disease pairs

Since most of the existing methods used pure linear or non-linear features for prediction, the complex relationships between miRNAs and diseases are difficult to express by single-category feature. In order to solve this problem, we fuse linear and non-linear features of nodes into a computational framework and combine them to perform our prediction task. Specifically, the linear feature is connected with the non-linear feature to respectively obtain the new feature vectors of miRNA and disease nodes:

15 $${\text{Mi}}_{{{\text{new\_feature}}}} = \;\left[ {F_{m} ,\;F^{\prime}_{m} } \right]$$

16 $${\text{Dis}}_{{{\text{new\_feature}}}} = \;\left[ {F_{d} ,\;F^{\prime}_{d} } \right]$$

where *F*_*m*_, *F*_*d*_ respectively denotes the linear features of miRNAs and diseases with feature dimension *l*. *F′*_*m*_, *F′*_*d*_ respectively denotes the non-linear features of miRNAs and diseases with dimension *l′*. Matrix *Mi*_*new*_*feature*_ ∈ *R *^*m*×(*l*+*l′*)^ denotes the *m* miRNA vectors with feature dimension (*l* + *l′*). Similarly, the matrix *Dis*_*new*_*feature*_ ∈ *R *^*n*×(*l*+*l′*)^ represents the *n* disease vectors with feature dimension (*l* + *l′*). Then we use *F*_*md*_ to denote the features of miRNA-disease pair (*i*, *j*) as follows:

17 $$F_{md} {(}{\text{i}} {,}{\text{j}} {)} = \left[ {f_{{m_{i} }} ,\;f^{\prime}_{{m_{i} }} ,\;f_{{d_{j} }} ,\;f^{\prime}_{{d_{j} }} } \right] \in R^{{2 \times \left( {l + l^{\prime}} \right)}}$$

### Predicting miRNA-disease relationship by RF

After deriving the features *F*_*md*_ of all miRNA-disease pairs, we capitalize on the random forest algorithm to construct the relationship prediction framework. Random forest is made up of multiple decision trees on the basis of bagging ensemble learning [68]. Each decision tree is trained and constructed by randomly selecting samples and sample features from the training dataset. Specifically, supposing that there are *N* samples in the training set, we apply the re-sample mode to randomly sample *N* samples to train a decision tree. During the training process of the decision tree, the algorithm first randomly selects *k*-dimensional features from the 2 × (*l* + *l′*)-dimensional features of samples. Then the selected *k*-dimensional features is used to guide the process of node splitting. Subsequently, we repeat the previous process *M* times to obtain *M* trained decision trees for constructing the corresponding random forests. Finally, predicted scores of the disease–miRNA pairs are determined by majority voting from the scores obtained by the *M* decision trees. The parametric experiment shows that our model achieves the best performance when the number of decision trees *M* is 350.

## Data Availability

The datasets and code as well as the predicted miRNA candidates of all diseases are provided on GitHub (https://github.com/ghli16/GATMDA).

## Abbreviations

miRNAs: MicroRNAs
GAT: Graph attention networks
TPR: True positive rate
FPR: False positive rate
ROC: Receiver operating characteristic
DAG: Directed acyclic graph
AUC: Area under ROC curve
CV: Cross validation
TCGA: The cancer genome atlas
RF: Random forest
Adaboost: Adaptive boosting
XGBoost: Extreme gradient boosting
Light GBM: Light gradient tree boosting machine
